# Oxidative stress and aberrant signaling in aging and cognitive decline

**DOI:** 10.1111/j.1474-9726.2007.00294.x

**Published:** 2007-06

**Authors:** Wulf Dröge, Hyman M Schipper

**Affiliations:** 1 Immunotec Research Ltd. 300 Joseph-Carrier, Vaudreuil-Dorion, Quebec, Canada J7V 5V5; 2 Department of Neurology and Neurosurgery and Department of Medicine (Geriatrics), McGill University, Centre for Neurotranslational Research, Lady Davis Institute for Medical Research S.M.B.D. Jewish General Hospital, Montreal, Quebec, Canada H3T 1E2

**Keywords:** oxidative brain aging, brain aging, aging, autophagy, cognitive aging, insulin signaling, sirtuins

## Abstract

Brain aging is associated with a progressive imbalance between antioxidant defenses and intracellular concentrations of reactive oxygen species (ROS) as exemplified by increases in products of lipid peroxidation, protein oxidation, and DNA oxidation. Oxidative conditions cause not only structural damage but also changes in the set points of redox-sensitive signaling processes including the insulin receptor signaling pathway. In the absence of insulin, the otherwise low insulin receptor signaling is strongly enhanced by oxidative conditions. Autophagic proteolysis and sirtuin activity, in turn, are downregulated by the insulin signaling pathway, and impaired autophagic activity has been associated with neurodegeneration. In genetic studies, impairment of insulin receptor signaling causes spectacular lifespan extension in nematodes, fruit flies, and mice. The predicted effects of age-related oxidative stress on sirtuins and autophagic activity and the corresponding effects of antioxidants remain to be tested experimentally. However, several correlates of aging have been shown to be ameliorated by antioxidants. Oxidative damage to mitochondrial DNA and the electron transport chain, perturbations in brain iron and calcium homeostasis, and changes in plasma cysteine homeostasis may altogether represent causes and consequences of increased oxidative stress. Aging and cognitive decline thus appear to involve changes at multiple nodes within a complex regulatory network.

## Introduction

Aging research is currently dominated by two competing concepts. The spectacular lifespan extension in certain mutants of nematodes, fruit flies, or mice suggested the possibility that aging may be a regulated process under the control of a few ‘aging genes’, notably genes involved in the insulin signaling pathway and in the expression of certain ‘silent information – regulating proteins’ or sirtuins (Kenyon *et al*., 1993; Dorman *et al*., 1995; Kimura *et al*., 1997; Hekimi & Guarente, 2003; Howitz *et al*., 2003; Sauve *et al*., 2006). On the other hand, there is a growing body of evidence supporting the ‘free-radical theory of aging’ (Harman, 1956), which predicts that the lifespan of an organism could be increased by augmenting antioxidant defenses. In this review, we summarize recent findings suggesting that the age-related increase in oxidative stress may have a direct impact on the insulin signaling pathway and downregulate sirtuin proteins. This review focuses mainly on the central nervous system although it is understood that many of the principles outlined may be germane to aging in non-neural tissues.

The decline in cognitive and motor functions is one of the hallmarks of ‘normal aging’ (Bartus, 1990; Kluger *et al*., 1997). It includes conspicuous changes in learning and memory, which have been linked to the hippocampus, as well as a decline in strength, balance, and coordination, which map to the basal ganglia and cerebellum (Joseph, 1992; Bickford, 1993; McDonald & White, 1994; Devan *et al*., 1996; Oliveira *et al*., 1997; Foster, 1999). In contrast to neurodegenerative diseases, the cognitive decline in ‘normal aging’ may not be associated with a significant loss of neurons (Gallagher *et al*., 1996).

## Evidence for an age-related increase in oxidative damage and decrease in antioxidant levels

The putative role of oxygen radicals and radical-derived reactive oxygen species (ROS) in neurodegeneration and cognitive decline has been reviewed previously (Gallagher *et al*., 1996; Berr *et al*., 2000; Serrano & Klann, 2004). The age-related increase in oxidative brain damage is best exemplified by products of lipid peroxidation (Gupta *et al*., 1991; Cini & Moretti, 1995; Murray & Lynch, 1998; O'Donnel & Lynch, 1998; Calabrese *et al*., 2004; Richwine *et al*., 2005; Zhu *et al*., 2006), protein oxidation (Forster *et al*., 1996; Mohsen *et al*., 2005; Sigueira *et al*., 2005; Poon *et al*., 2006), and oxidative modifications in nuclear and mitochondrial DNA (Hamilton *et al*., 2001). An increase in protein carbonyl levels has been demonstrated for various brain regions including the hippocampus (Mohsen *et al*., 2005, Siqueira *et al*., 2005).

Age-related memory impairment is correlated with a decrease in brain and plasma antioxidants (Perrig *et al*., 1997; Perkins *et al*., 1999; Berr *et al*., 2000; Rinaldi *et al*., 2003). Intracellular glutathione (GSH) concentration as well as the ratio of glutathione : glutathione disulfide (GSH : GSSG ratio) were found to decrease with age in different animal models (Chen *et al*., 1989; Iantomasi *et al*., 1993; Oubidar *et al*., 1996; Pallardo *et al*., 1999; Sasaki *et al*., 2001; Liu, 2002; Sandhu & Kaur, 2002; Rebrin *et al*., 2003; Wang *et al*., 2003; Suh *et al*., 2004, 2005). This decrease in GSH level and/or GSH : GSSG ratio was found in all mammalian brain regions tested including the hippocampus (Calabrese *et al*., 2004; Balu *et al*., 2005; Donahue *et al*., 2006; Zhu *et al*., 2006). As cysteine is the most limiting precursor for intracellular GSH biosynthesis, it is reasonable to hypothesize that the decrease in intracellular GSH may be mechanistically related to the age-related decrease in post-absorptive plasma cysteine that has been found in humans between the third and the ninth decade of life (Hack *et al*., 1998). Most cells and tissues have relatively strong membrane transport activity for reduced cysteine and weak or no transport activity for its relatively large oxidized derivative, cystine.

## Age-related derangement of redox-regulated signals

As ROS production by various tightly regulated NAD(P)H oxidase, isoenzymes is known to play a role in many redox-sensitive signaling pathways, any increase in ROS levels and/or any oxidative shift in the thiol-disulfide redox status may cause a shift in the set points of numerous physiological processes (reviewed in Dröge, 2002). The age-related increase in the steady state level of interleukin 6 (IL-6) mRNA and IL-6 production in the brain (Ye & Johnson, 1999; Richwine *et al*., 2005) is only one case in point. *In vivo* and *in vitro* studies indicate that this increase results from enhanced binding of the redox-sensitive transcription factor nuclear factor κ B (NF-κB) to the IL-6 promoter in microglial cells (Ye & Johnson, 1999, 2001). The age-related increase in IL-6 is reminiscent of the systemic increase in the concentration of the inflammatory cytokine, tumor necrosis factor α (TNF-α), which is also controlled by NF-κB. The increase in TNF-α was ameliorated by treatment with the GSH prodrug N-acetyl cysteine (NAC) (reviewed in Dröge, 2005b).

## Redox sensitivity of the insulin signaling pathway and the role of autophagy and sirtuin proteins

**Fig. 1:**
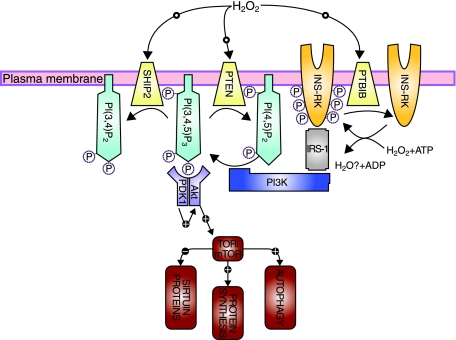
Oxidative upregulation of the insulin receptor signaling cascade in the absence of insulin. Akt, serine/threonine kinase PKB; IRS-1, insulin-receptor substrate; INS-RK, insulin receptor tyrosine kinase; PDK1, phosphoinositide-dependent protein kinase-1; PI3K, phosphatidylinositol-3 kinase; PTEN, phosphatase and tensin homolog on chromosome 10; PTP1B, protein tyrosine phosphatase 1B; PI(3,4)P_2_, phosphatidylinositol(3,4) diphosphate; PI(4,5)P_2_, phosphatidylinositol(4,5) diphosphate; PI(3,4,5)P_3_, phosphatidylinositol (3,4,5) triphosphate; SHIP2, SH2-domain-containing inositol phosphatase; TOR/mTOR, (mammalian) target of rapamycin. The question mark indicates that H_2_O as a product of this reaction has not been formally proven (for other details see text).

The insulin receptor is expressed throughout the brain, including the hippocampus (Dou *et al*., 2005; Wada *et al*., 2005; Benedict *et al*., 2006). The importance of the insulin signaling pathway in aging has been convincingly demonstrated by genetic studies of nematodes, fruit flies, and mice. In all of these species, a dramatic lifespan extension was often associated with an impairment of the insulin receptor signaling pathway or with an increased expression of sirtuin family proteins, which are negatively regulated by this signaling cascade (Kenyon *et al*., 1993; Dorman *et al*., 1995; Kimura *et al*., 1997; Hekimi & Guarente, 2003, Howitz *et al*., 2003; Rogina & Helfand, 2004; Sauve *et al*., 2006). A substantial lifespan extension of *Caenorhabditis elegans*, for example, was achieved by a mutation in *daf-2*, which encodes an insulin receptor ortholog. Lifespan was negatively affected by the target of rapamycin (TOR), which functions downstream in the insulin receptor signaling pathway (Kapahi *et al.*, 2004). By activating TOR or its mammalian counterpart mTOR, the insulin receptor signaling pathway downregulates the expression of proteins of the sirtuin family and the autophagic mechanism of proteolysis (schematically illustrated in Fig. 1; reviewed in Dröge, 2005a).

The autophagic mechanism of proteolysis plays an important role in the maintenance of cellular integrity by removing (damaged) mitochondria and other organelles (reviewed in Dröge, 2003). Not unexpectedly, autophagy was shown to be required for lifespan extension in longevity mutants of *C. elegans* (Melendez *et al*., 2003). Mice genetically engineered to lack either of the autophagy genes *Atg5* or *Atg7* in neuronal cells during later stages of embryogenesis develop signs of neurodegeneration and eventually show inclusion bodies akin to those seen in various human aging-related neurodegenerative disorders (Hara *et al*., 2006; Komatsu *et al*., 2006). In line with this finding, there is ample evidence implicating proteolytic stress in a wide range of neurodegenerative diseases (Ciechanover & Brundin, 2003; Bossy-Wetzel *et al*., 2004). Emerging evidence supports the view that induction of autophagy is a neuroprotective response and that inadequate or defective authophagy, rather than excessive autophagy, promotes neuronal cell death in most neurodegenerative disorders (Nixon, 2006; Rubinsztein, 2006). Signs of excessive autophagy have been reported in patients with Alzheimer's disease (AD) but are uncommon in brains devoid of AD pathology (Nixon *et al*., 2005).

Sirtuin proteins are a family of protein deacetylases, which regulate a diverse set of pathways implicated in the aging process (reviewed in Hekimi & Guarente, 2003). Certain sirtuins were found to regulate glucose and fat metabolism in mammals (Guarente & Picard, 2005; Rodgers *et al*., 2005) and to enhance mitochondrial biogenesis in liver and muscle through the transcriptional coactivator peroxisome proliferator-activator receptor-γ coactivator 1α (PGC-1α) (Rodgers *et al*., 2005; Lerin *et al*., 2006), and cell survival by deactivating the tumor surpressor, p53 (Smith, 2002). In view of these multiple effects, the exact role of sirtuin proteins in the aging process remains obscure. The plant-derived polyphenol, resveratrol, was found to increase the catalytic (deacetylase) activity of certain sirtuin proteins and to extend the lifespan of yeast by 70% (Howitz *et al*., 2003). In the short-lived fish, *Nothobranchius furzeri*, resveratrol extended the maximum lifespan by 60%, delayed the decay of locomotor activity and cognitive performance, and attenuated neurofibrillary degeneration in the brain (Valenzano *et al*., 2006). Middle-aged mice on a high-calorie diet that were treated with resveratrol for the remainder of their life showed a significantly increased lifespan, increased postprandial insulin sensitivity, AMP-activated protein kinase (AMPK), and PGC-1α activity, increased mitochondrial numbers, and improved motor function (Baur *et al*., 2006). Whether sirtuin proteins similarly influence human aging is not known.

**Fig. 2:**
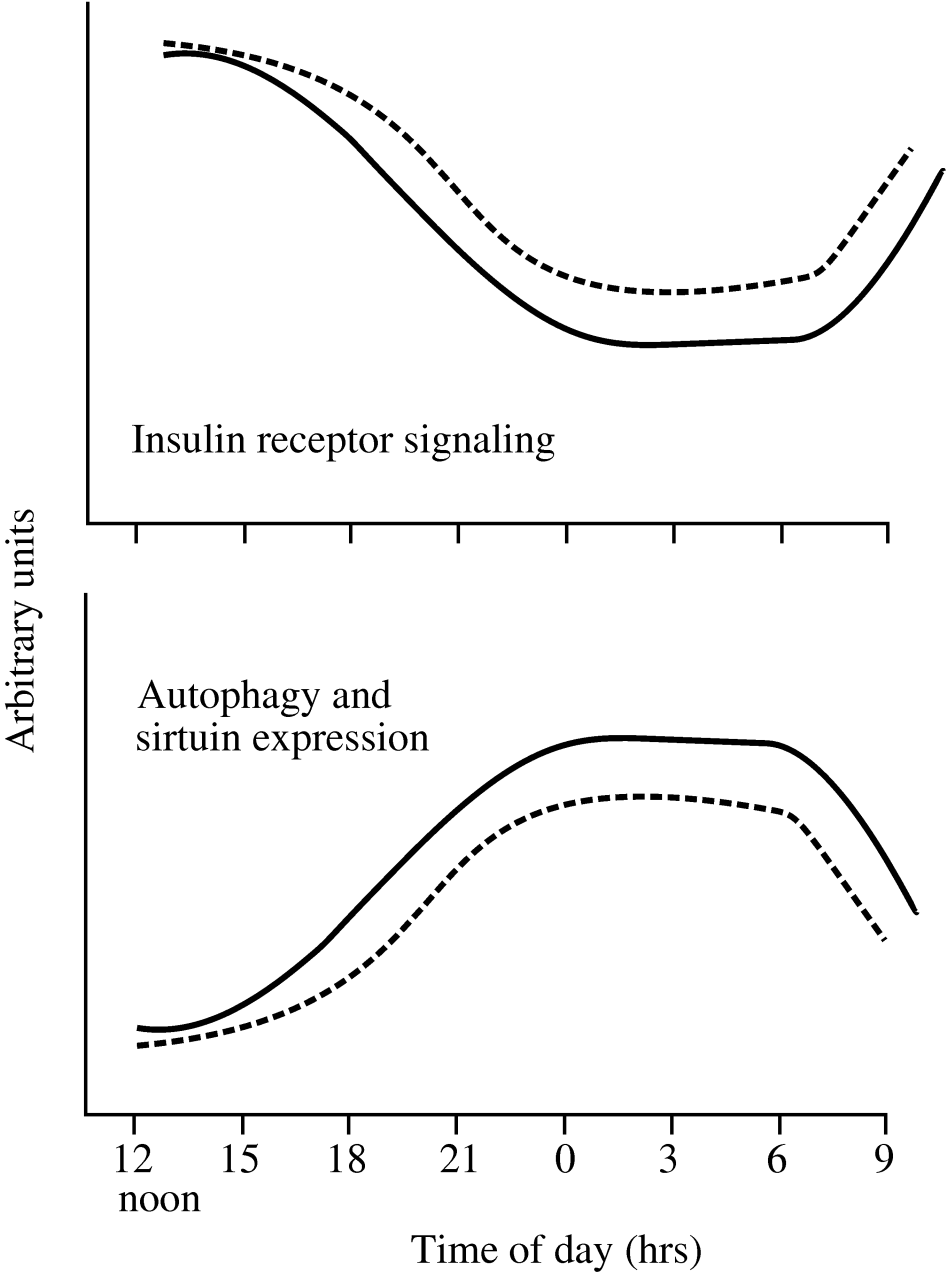
Interrelated changes of insulin receptor signaling, autophagy, and sirtuin expression during the day (hypothetical scheme). The solid lines illustrate the temporal changes in young healthy subjects. During the night, that is, in the post-absorptive state, insulin receptor signaling is low and autophagy and sirtuin activity are accordingly high. In the aging organism (dashed lines), the age-related oxidative shift in redox status leads to an increased basic signaling activity from the insulin receptor in spite of low insulin levels; autophagy and sirtuin expression are accordingly suppressed.

**Fig. 3:**
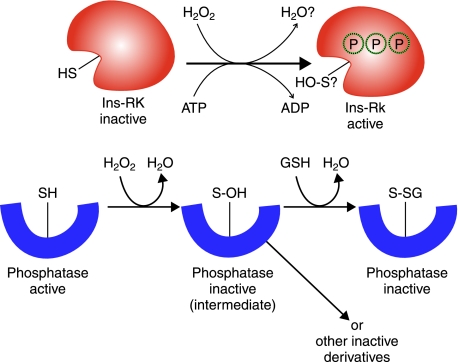
Oxidative activation of insulin receptor kinase and inactivation of phosphatases (schematic illustration). Upper panel: in the absence of insulin, basic insulin receptor kinase (Ins-RK) activity is relatively low. Hydrogen peroxide oxidizes cysteine sulfhydryl (SH) groups into sulfenic acid moieties (S-OH). This oxidative process allows adenosine 5’-triphosphate (ATP) to phosphorylate the insulin receptor kinase at its catalytic site and to render the kinase catalytically active. Lower panel: phosphatases typically contain an SH group at their catalytic center. Upon oxidation by hydrogen peroxide the phosphatase is converted into a catalytically inactive intermediate and other inactive derivatives. The question marks indicate that these reaction products have not been formally proven.

As autophagy and sirtuin expression are down-regulated by the insulin signaling cascade, it is inferred that autophagy and sirtuin activity are optimally expressed when circulating insulin levels are low, that is, in the post-absorptive state during the night (see Fig. 2). This important point remains to be confirmed. The insulin-independent *basic* activity of the insulin receptor signaling pathway (i.e. its activity in the post-absorptive state during the night) is weak but subject to redox regulation (see Fig. 1). The signaling cascade is negatively controlled by several phosphatases, including protein tyrosine phosphatase 1B (PTB 1B), phosphatase and tensin homolog on chromosome 10 (PTEN) and SH2-domain-containing inositol phosphatase (SHIP2), all of which are inactivated under moderately oxidative conditions. In addition, the *basic* insulin receptor tyrosine kinase (Ins-RK) activity is strongly increased by low concentrations of hydrogen peroxide or by an oxidative shift in the GSH redox status (Schmitt *et al*., 2005). In the presence of adenosine 5’-triphosphate (ATP) and hydrogen peroxide, the insulin receptor kinase domain is phosphorylated at its catalytic site and thereby rendered catalytically active in the absence of insulin (schematically illustrated in Fig. 3). The phosphatases, in turn, contain a redox-sensitive cysteine moiety in their catalytic site, which is converted by hydrogen peroxide into a sulfenic acid moiety, which renders the phosphatase inactive (Fig. 3). The balance between kinase and phosphatase activities determines the rate of phosphorylation of the insulin receptor kinase domain and several downstream targets including the phosphatidylinositol phosphates, the serine/threonine kinase Akt1 and the TOR. Ultimately, this balance controls the aging-related functions of autophagy and sirtuin proteins (Fig. 1). The redox sensitivity of the *basic* insulin receptor signaling activity *in vivo* has been confirmed in a clinical study of nondiabetic obese persons in whom basic (i.e. post-absorptive) insulin receptor signaling was decreased after supplementation with relatively small doses of NAC (Hildebrandt *et al*., 2004). It is therefore conceivable that autophagic proteolysis and sirtuin activity may be compromised by the oxidative conditions that prevail in old age as schematically illustrated in Fig. 2. This important prediction remains to be confirmed.

## Effects of GSH enhancing antioxidants on oxidative damage and functional decline – suggestive evidence for a cause–effect relationship

N-acetyl cysteine is widely used both in clinical medicine and in experimental animal studies to increase the availability of cysteine and intracellular GSH, that is, the quantitatively most important cellular antioxidant. Feeding of NAC-containing pellets (0.3% w/w) to 48-week-old mice for 24 weeks caused a significant decrease in protein carbonyls in synaptic mitochondria of the brain in comparison with untreated controls (Banaclocha *et al*., 1997). Chronic dietary administration of NAC was also shown to preserve mitochondrial proteins involved in oxidative phosphorylation in the liver of senescent mice (Miquel *et al*., 1995). Feeding of NAC-containing pellets (0.3% w/w) to mice from 12 months until killed at 28 months resulted in a significant increase in ADP-stimulated respiration in brain mitochondria. In addition, NAC treatment reversed the age-related decline in cytochrome *c* content and the decrease in cytosolic GSH in the brain (Cocco *et al*., 2005).

The age-related decline in memory function was also ameliorated by NAC in a recent study on rats. Again, this was associated with diminished levels of protein oxidation and lipid peroxides in brain synaptic mitochondria (Martinez *et al*., 2000). Furthermore, a diet containing 7.2 mg NAC per day significantly attenuated the accelerated, age-related cognitive dysfunction observed in transgenic mice overexpressing growth hormone (Lemon *et al*., 2003). The excitatory amino acid carrier 1 (EAAC1) serves as a transporter for glutamate, aspartate and reduced L-cysteine. Although EAAC1 is not the only cysteine transporter, EAAC1 deficient mice (EAAC1^−/–^) have relatively low neuronal (hippocampal) GSH levels and, with advancing age, develop brain atrophy and behavioral changes. Increasing cysteine availability in these animals by intraperitoneal injections of 150 mg kg^−1^ of NAC normalized brain GSH concentrations and decreased the extent of neuronal death after oxidant exposure (Aoyama *et al*., 2006). Collectively, these data attest to the important contribution of the thiol redox status in the preservation of healthy cognition in aging rodents.

(R)-α-Lipoic acid (LA) is a coenzyme involved in mitochondrial redox processes. The reduced form of LA chelates iron and copper and recycles several antioxidants including GSH and vitamins C and E (Packer *et al*., 1997; Moini *et al*., 2002). Feeding of LA (0.2% w/w for 2 weeks) to 24- to 28-month-old rats reversed the age-related increase in iron levels and the age-related decrease in GSH and ascorbic acid levels in the brain (Suh *et al*., 2005). Several reports described positive effects of LA in combination with acetyl-L-carnitine on memory and brain mitochondrial functions in old rats (Liu *et al*., 2002a,b). Rats that were fed LA exhibited improved spatial learning and memory (Stoll *et al*., 1994).

## Other experimental strategies to ameliorate oxidative damage

The radical scavenger α-phenyl-tert-butyl-nitrone (PBN) has potent salutary effects in certain aging-related diseases (reviewed in Floyd *et al*., 2002). Intraperitoneal administration of PBN in aged gerbils for 14 days caused a significant decrease in brain levels of oxidized protein and improved performance in a temporal and spatial memory test when compared to age-matched controls. The same treatment had no effect on young adult gerbils (Carney *et al*., 1991). Similar results have been obtained in mice (Fredriksson & Archer, 1996). Chronic systemic administration of synthetic catalytic scavengers of ROS (i.e. superoxide dismutase/catalase mimetics) to mice for 3 months between 8 and 11 months of age almost completely reversed not only the increase in brain oxidative damage but also the cognitive deficits (Liu *et al*., 2003). It remains to be determined whether these antioxidative treatments may have a GSH sparing effect and indirectly elevate cellular GSH levels.

Supportive evidence for a role of oxidative stress in aging has also been obtained in animal studies of calorie restriction. A restriction of dietary calories results in significant lifespan extension in diverse organisms (Sohal & Weindruch, 1996; Weindruch & Walford, 1997). In several independent studies of mice, rats, and fruit flies, the increased survival of calorically restricted animals was associated with a decrease in tissue oxidative damage (Hamilton *et al*., 2001; Sohal *et al*., 1994; Zheng *et al*., 2005). Also, in a series of long-lived strains of worms, flies, and mice, the increased lifespan was correlated with increased oxidative stress resistance (Orr & Sohal, 1994; Lin *et al*., 1998; Parkes *et al*., 1998; Honda & Honda, 1999; Lee *et al*., 1999; Migliaccio *et al*., 1999; Taub *et al*., 1999). Again, GSH levels have not been reported.

## Putative mechanisms accounting for the age-related increase in oxidative damage: changes in antioxidant enzymes

Several authors suggested the possibility that the increase in oxidative stress may result, at least partly, from a relative decrease in antioxidant enzyme activities (Tian *et al*., 1998; Navarro *et al*., 2004; Sigueira *et al*., 2005). A decline in GSH peroxidase activity has been found in hippocampus and hypothalamus of aged rats (Sigueira *et al*., 2005). A decrease in the activities of Mn and CuZn superoxide dismutase isoenzymes and catalase has been found in the brain, heart, liver, and kidney of aging mice (Gupta *et al*., 1991; Navarro *et al*., 2004). A decrease in the apparent catalytic turnover of γ-glutamyl cysteine ligase (GCL) and/or GCL subunit expression has been found in old rats (Liu, 2002). However, it should be noted that an age-related decline in brain antioxidant enzyme activities has not been confirmed by other investigators and remains controversial (Serrano & Klann, 2004).

## Oxidative mitochondrial damage as part of a *vicious cycle* leading to increased ROS production

Mitochondria are widely believed to play a key role in aging because they are a major source of oxygen radicals and arguably their most important targets. Mitochondrial DNA is particularly vulnerable to oxidative damage and shows a more than ten-fold greater mutation rate than nuclear DNA. Mutated mitochondrial DNA may code for abnormal cytochromes and may cause infidelity of the electron transport chain associated with increased superoxide radical production and a *vicious cycle* of progressively increasing oxidative stress (Linnane *et al*., 1992; Finkel & Holbrook, 2000; Sohal *et al*., 2002; Hekimi & Guarente, 2003; Sastre *et al*., 2003; Vina *et al*., 2003; Navarro & Boveris, 2004; Schipper, 2004; Balaban *et al*., 2005). Moreover, accumulation of peroxidation products in mitochondria leads to a decrease in ATP production and compromises the maintenance of cellular homeostasis (Chance *et al*., 1979). Mitochondrial dysfunction and mitochondria-derived ROS have been implicated in both normal brain aging and neurodegenerative diseases (Toescu & Verkhratsky, 2003; Lin & Beal, 2006). A study of rat brain mitochondria also revealed an age-related increase in mitochondrial fragility, volume, and water permeability (Navarro & Boveris, 2004). In the mammalian central nervous system (CNS), cellular senescence is often associated with the accumulation of mitochondria-derived cytoplasmic inclusions such as Gomori-positive glial granules and corpora amylacea (Brunk & Terman, 2002; Schipper, 2004). Evidence supporting the free radical-mitochondrial theory of aging has accrued from a broad range of *in vitro* studies and whole animal models from unicellular organisms to mammals. However, the theory is not immune from controversy and some investigators maintain that aging exerts no adverse effect on either the electron transport chain or oxidative phosphorylation (Maklashina & Ackrell, 2004).

## Dysregulation of iron homeostasis as a potential cause of oxidative stress

Free iron is a potential source of oxidative stress because it catalyses the conversion of hydrogen peroxide into highly reactive hydroxyl radicals by the Fenton reaction. In addition, iron-dependent lipid peroxidation may also generate potentially toxic peroxyl/alkoxyl radicals (Gutteridge, 1982), and ferrous free iron may convert neutral catechols (e.g. dopamine) to neurotoxic o-semiquinone intermediates (Schipper, 2004). An age-related increase in iron content has been found in human brain tissue (Martin *et* *al*., 1998) as well as rat and mouse brain (Cook & Yu, 1998; Sohal *et al*., 1999; Donahue *et al*., 2006). In rats the concentrations of several metals, including iron, increase in the hippocampus and frontal cortex (Donahue *et al*., 2006). In the aging human brain, excessive iron accumulation associated with markers of free radical injury and mitochondrial DNA deletion are most prominent in the basal ganglia, hippocampus, and certain cerebellar nuclei (Soong *et al*., 1992). In rat astrocytes hydrogen peroxide was found to induce the heme-degrading enzyme heme oxygenase 1 (HO-1), which fosters the liberation of free cytosolic Fe^++^ (Mydlarski *et al*., 1993). HO-1 may therefore exacerbate brain aging processes by engaging in a neuropathological *vicious cycle* of ROS generation (Schipper, 2004). The most common cell type in the brain to stain for iron under normal conditions is the oligodendrocyte (Connor & Menzies, 1999). In humans, an age-related increase in iron staining and ferritin immunoreactivity was found in microglia and astrocytes in various brain regions including the hippocampus (Zecca *et al*., 2004). Thus, aging-related oxidative stress may participate in a *vicious cycle* of events characterized by dysregulation of cellular iron homeostasis, mitochondrial insufficiency, bioenergetic failure and augmented ROS generation by the electron transport chain. Iron chelators, free radical scavengers and inhibitors of glial HO-1 expression, alone or in combination, may be useful in the management of aging-related human neurodegenerative afflictions (Schipper, 2004). The observation that brain iron levels can be reduced in old rats by oral administration of the blood-brain barrier-permeable chelator, LA (Suh *et al*., 2005) may be an important step in this direction.

## Dysregulation of calcium homeostasis and oxidative stress

Another potential *vicious cycle* may involve changes in the cytosolic and mitochondrial calcium concentration (Toescu & Verkhratsky, 2003; Foster, 2006). Well-controlled temporary elevations of cytosolic calcium concentrations play a role in numerous cellular signaling systems and are typically induced by the release of calcium from the endoplasmic reticulum (ER) (reviewed in Toescu & Verkhratsky, 2003). A growing body of evidence suggests that the calcium channels, which control calcium efflux from the ER in response to different biochemical signals, are also sensitive to small changes in ROS concentrations or changes in the intracellular thiol redox status, suggesting that these calcium channels serve as physiological redox sensors (Okabe *et al*., 2000; Pessah & Feng, 2000; reviewed in Camello-Almaraz *et al*., 2006; Zima & Blatter, 2006). Effects of hydrogen peroxide on calcium fluxes have been demonstrated in rat hippocampal cells, cortical astrocytes, and dentate granule cells (Jacobson & Duchen, 2002; Akaishi *et al*., 2004; Gonzalez *et al*., 2006). Robust elevation of cytosolic calcium causes calcium flux into the mitochondrial matrix, which, in turn, enhances mitochondrial ROS production, thereby completing the *vicious cycle* (Van de Water *et al*., 1994; Bindokas *et al*., 1996). Increased mitochondrial calcium concentrations combined with increased ROS production are characteristic of neurodegenerative conditions, such as AD and ischemic brain injury (Toescu & Verkhratsky, 2003; Starkov *et al*., 2004). It has been suggested that calcium-dependent mitochondrial ROS production may also play a role in normal cellular physiology (see Hongpaison *et al*., 2003). There is a strong possibility that the age-related oxidative shift in redox status may cause subpathological changes in calcium homeostasis and especially changes in the kinetics of calcium fluctuations (see Toescu & Verkhratsky, 2003). Whether these, in turn, may have a substantial effect on mitochondrial ROS production remains to be ascertained.

## Age-related decrease in plasma cysteine concentration

Neurons exhibit relatively strong membrane transport activity for the small amino acid, cysteine but weak transport activity for its larger oxidized derivative cystine. Therefore, and because the plasma concentration of reduced cysteine is extremely low in comparison to most other amino acids, cysteine is the limiting amino acid for GSH biosynthesis in neural and most other cells (Bannai & Tateishi, 1986; Aoyama *et al*., 2006). Clinical studies have shown that the post-absorptive plasma cysteine concentration in healthy human subjects decreases significantly between the third and ninth decade of life (Hack *et al*., 1998; Dröge, 2005b). Supplementation with NAC or a cysteine-rich protein has been shown to improve muscular strength and other functional and biochemical parameters, which are typically compromised in old age (Hauer *et al*., 2003; reviewed in Dröge, 2005a,b).

Why the 70-year-old seemingly healthy subject has, on average, a substantially lower post-absorptive plasma concentration of reduced cysteine than a 20-year-old person with the same dietary protein intake remains enigmatic. The basic biochemical mechanisms of cysteine biosynthesis, utilization, and catabolism are well known, and yet the mechanisms responsible for the age-related changes in cysteine homeostasis remain obscure. These changes may be related to the facts that (i) in the postprandial state, a substantial proportion of the dietary cysteine is normally converted into hepatic GSH and proteins, which serve as a reservoir for cysteine (Cho *et al*., 1984; Cheema-Dhadli & Halperin, 1993), and (ii) the rate of postprandial protein synthesis per time unit decreases with age (Volpi *et al*., 2000; Dardevet *et al*., 2002; Combaret *et al*., 2005; Fujita & Volpi, 2006). As the hepatic concentration of free cysteine is a key regulator of its own catabolism into sulfate (Stipanuk *et al*., 1992; Lee *et al*., 2004), we tentatively assume that the post-absorptive cysteine concentration in old age decreases as a consequence of the age-related decrease in postprandial protein synthetic capacity and the resulting failure to rapidly clear dietary cysteine before it is catabolized into sulfate. To understand and to reverse these changes in cysteine homeostasis may be a worthwhile and achievable goal in the near future.

## Conclusions

There is an impressive body of evidence (i) for an age-related increase in oxidative stress in both humans and experimental animals, (ii) for the redox sensitivity of the insulin signaling cascade as demonstrated by numerous molecular studies, and (iii) for the dramatic impact of the insulin receptor signaling cascade and insulin-controlled sirtuin proteins on the lifespan of nematodes, fruit flies, and mice as demonstrated by genetic studies. There is therefore a strong possibility that oxidative enhancement of the insulin receptor signaling pathway may have an even greater impact on aging than the direct oxidative damage to structural constituents. In spite of the persuasive mosaic of data, there are still many information gaps especially regarding the extrapolation of animal data to humans. With regard to insulin-controlled, aging-related functions, there is suggestive evidence that inadequate autophagic proteolysis does play a role including in human aging, but we still do not know whether sirtuins or resveratrol are capable of influencing fundamental aging processes in humans. Also, the predicted effects of the age-related oxidative stress on autophagy and sirtuin activity as well as the corresponding putative effects of antioxidants have not yet been demonstrated experimentally.

The notion that oxidative stress plays a causative role in age-related functional decline is not proven but it is already strongly supported by the effects of antioxidants on various correlates of aging. This review has been somewhat biased in favor of thiol containing and GSH enhancing antioxidants and may not have done justice to other types of antioxidants and ROS scavengers. One of the reasons for this bias is the large number of reports concerning the effects of NAC and the thiol compound α-LA. In patients with AD, NAC was shown to improve certain cognitive tasks (Adair *et al*., 2001). In our view, understanding and reversing age-related changes in plasma thiol (cysteine) homeostasis is an achievable goal with important implications for the amelioration of a broad spectrum of neurological and systemic conditions linked to the aging process.

The abundant evidence for an age-related increase in products of oxidative damage in the mammalian brain is in good agreement with findings in other tissues (reviewed in Beckman & Ames, 1998; Dröge, 2002). ROS-mediated mitochondrial damage, the increase in iron deposition, dysregulation of Ca^++^ homeostasis and post-absorptive plasma cysteine homeostasis, and impaired autophagy and sirtuin activity secondary to aberrant insulin receptor signaling may represent several pivotal mechanisms in a *vicious cycle* leading to progressively increasing intracellular ROS concentrations and oxidative stress.

**Fig. 4:**
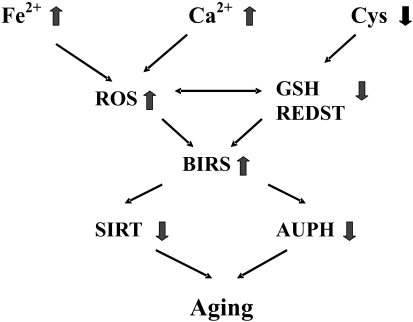
Pathomechanisms of aging: deregulation at key ‘nodes’ in a network of interrelated physiological processes. AUPH, autophagic mechanism of proteolysis involved in the turnover of (damaged) mitochondria and other cellular constituents. Autophagy is negatively regulated by insulin. BIRS, basal activity of the insulin receptor signaling pathway, that is, in the post-absorptive state.Ca^2+^, mitochondrial calcium concentrations (calcium homeostasis); Cys, post-absorptive plasma cysteine concentration (cysteine homeostasis); Fe^2+^, nontransferrin iron concentration (iron homeostasis); GSH, intracellular glutathione concentrations; REDST, glutathione redox status; ROS, intracellular reactive oxygen species; SIRT, sirtuin proteins impact lifespan and are negatively regulated by insulin.

Taken together, the evidence suggests that aging is not mediated by a single gene or by a single ‘master mechanism’ of oxidative damage but may rather result from progressive dysregulation in a complex functional network as illustrated schematically in Fig. 4. In thermodynamic terms this reflects an increase in entropy. Similar conclusions were recently expressed by Drachman (2006) with respect to the multiplicity of cellular and molecular mechanisms that contribute to senescence in the human brain. Certain genes or certain biochemical processes may, nevertheless, contribute more than others. If this concept is basically correct, a cocktail of multiple interventions that simultaneously targets several of the most important nodes in this network may be required to limit age-related dyshomeostasis.
